# High-resolution functional photoacoustic monitoring of vascular dynamics in human fingers

**DOI:** 10.1016/j.pacs.2021.100282

**Published:** 2021-06-25

**Authors:** Joongho Ahn, Jin Young Kim, Wonseok Choi, Chulhong Kim

**Affiliations:** Departments of Electrical Engineering, Convergence IT Engineering, Mechanical Engineering, and Medical Device Innovation Center, Pohang University of Science and Technology, Pohang, 37673, Republic of Korea

**Keywords:** Photoacoustic microscopy, Blood vessel, Hemodynamics, Pulsation, Perfusion, Oxygen saturation, Digital vasculature

## Abstract

Functional imaging of microvascular dynamics in extremities delivers intuitive information for early detection, diagnosis, and prognosis of vascular diseases. High-resolution and high-speed photoacoustic microscopy (PAM) visualizes and measures multiparametric information of microvessel networks *in vivo* such as morphology, flow, oxygen saturation, and metabolic rate. Here, we demonstrate high-resolution photoacoustic monitoring of vascular dynamics in human fingers. We photoacoustically monitored the position displacement of blood vessels associated with arterial pulsation in human fingers. Then, during and after arterial occlusion, we photoacoustically quantified oxygen consumption and blood perfusion in the fingertips. The results demonstrate that high-resolution functional PAM could be a vital tool in peripheral vascular examination for measuring heart rate, oxygen consumption, and/or blood perfusion.

## 1 Introduction

An intricate and extensive network of vessels channels blood throughout the entire human body [1]. The blood circulating in the vessels transports oxygen and nutrients to cells and organs [2], so the vessels’ physical condition and circulatory ability are closely related to bodily dysfunctions. Insufficient delivery of oxygen or nutrients negatively affects tissues, impairing physiological functions [3]. Notably, the microvessels in the extremities, such as the fingers, and toes, are connected to the heart through macrovessels, and abnormalities of these macrovessels or of the heart itself can cause serious abnormalities of the microvessels [4]. When blood is not properly supplied to an extremity, it is exposed to vascular diseases and, in severe cases, may ultimately require amputation [5].

Different imaging techniques have been used to assess the structure and functioning of blood vessels. Both computed tomography (CT) angiography and magnetic resonance angiography provide wide-field images of primary arteries with high contrast [6,7]. However, these methods require ionizing radiation and/or contrast agents that are potentially harmful to some organs. Ultrasound (US) imaging, an agent-free and real-time imaging modality, provides anatomical information about the body, showing flow in blood vessels by using the Doppler effect [8]. However, all the above methods have low imaging resolution and so cannot diagnose impaired microcirculation [9]. Nailfold videocapillaroscopy (NVC) can image the capillaries with high resolution and observe blood flow at the microvessel level, but it can be applied only to the capillary loop of cuticles [10,11]. In addition, NVC cannot provide the oxygen saturation (sO_2_) of the microvessel, a key diagnostic indicator. Optical coherence tomography angiography (OCTA) can visualize the blood vessels by capturing more signal changes from the vessels over time than from static tissues [12]. OCTA can also estimate the flow and sO_2_ of the microvessels by using the Doppler effect and visible light spectroscopy [13,14]. However, although sO_2_ measurement using OCTA has been reported in human eye vessels [15], it has not yet been measured through the skin, due to the modality’s limited penetration depth [12]. Even when light passes through blood vessels and then bounces back from the tissues, OCTA registers the signal changes created by the blood vessels, which creates “false” blood vessels called OCTA projection artifacts that can affect clinical assessment [16].

As a superior alternative, photoacoustic (PA) imaging can directly visualize blood vessels because the hemoglobin in the blood is an endogenous absorber that generates PA waves [17]. Because oxyhemoglobin (HbO_2_) and deoxyhemoglobin (Hb) exhibit different absorption coefficients at different optical wavelengths, a spectroscopic technique can be applied to PA imaging to estimate sO_2_ [18]. In addition, because sound travels more slowly than light in tissue, depth-resolved 2D images can be created, stacks of which can be reconstructed to form 3D rendered images [19]. Exploiting the merits of the PA vascular imaging, previous studies have investigated structural and functional features such as vasomotion, sO_2_, and blood flow [[20], [21], [22]]. In addition, the recent development of high-repetition-rate pulsed lasers and high-speed scanners has significantly improved the imaging speed of photoacoustic microscopy (PAM) [[23], [24], [25], [26], [27], [28]]. Thanks to these advances, multi-wavelength and high-speed PAM has been developed and utilized in preclinical and clinical studies of blood vessels [[29], [30], [31], [32], [33], [34], [35], [36], [37], [38], [39]].

In this study, we use a dual-wavelength high-speed PAM system to capture the structural and functional dynamics of the micro-vessels in human fingers. We continuously imaged the 2D cross-sectional plane of the fingertips and found the position displacement of the blood vessels associated with arterial pulsation. Further, we monitored oxygen consumption during brachial cuffing and then observed blood perfusion after releasing the cuff. The results show that high-speed, high-resolution PAM can be invaluable in evaluating the vascular dynamics in human extremities.

## 2 Materials and methods

### 2.1 High resolution, high-speed, spectroscopic photoacoustic microscopy

**Fig. 1:**
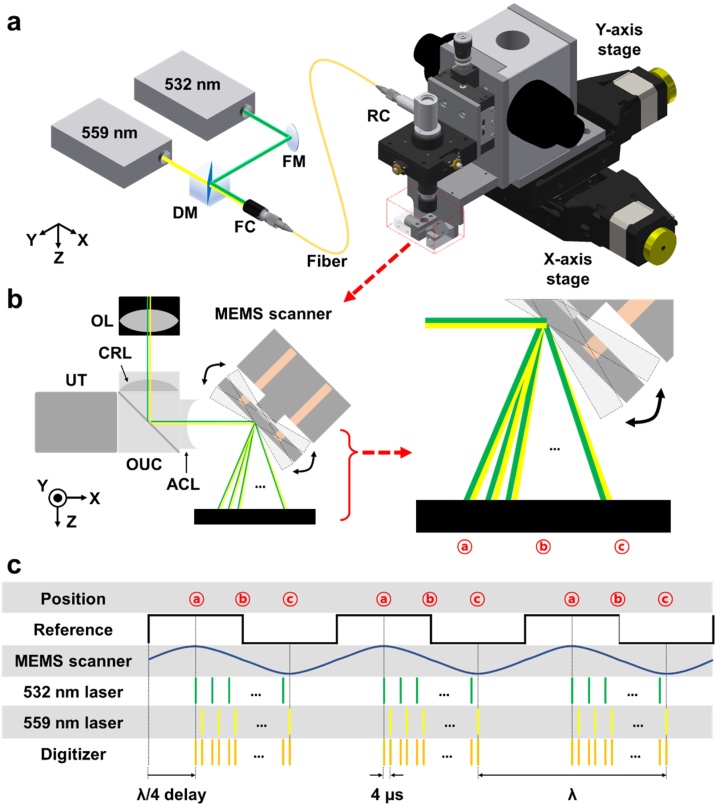
(a) Configuration of the high-speed optical-resolution photoacoustic microscopy (OR-PAM) system. (b) Optical paths of the two lasers as the MEMS scanner moves. (c) Fully synchronized timing diagram. MEMS, microelectromechanical system; FM, flat mirror; DM, dichroic mirror; FC, fiber collimator; RC, reflective collimator; OL, objective lens; UT, ultrasound transducer; CRL, correction lens; OUC, opto-ultrasound beam combiner; ACL, acoustic lens.

Fig. 1a shows the configuration of the high-resolution, high-speed, dual-wavelength PAM system, modified from a commercial high-speed PAM (OptichoM, Opticho, Republic of Korea). The system is equipped with two nanosecond pulsed lasers (AWAVE532-1W-10 K and MPA559, Advanced Optowave, NY, USA), which have 532 nm and 559 nm wavelengths, respectively. From the isosbestic point of 545 nm at which the absorption coefficients of oxyhemoglobin (HbO2) and deoxyhemoglobin (Hb) are equal, HbO2 absorbs 532 nm light more than Hb, and Hb absorbs 559 nm more than HbO2. Using the two wavelengths, the PAM can measure oxygen saturation using a spectral unmixing method [22]. The two laser beams are combined by a dichroic mirror (DMSP550R, Thorlabs, NJ, USA), but first the 532-nm beam is attenuated by a continuously variable neutral density (ND) filter wheel (NDC-50C-4 M, Thorlabs, NJ, USA) to balance the optical power of the two beams. The combined beam is coupled to a single mode fiber (P1-460B-FC-1, Thorlabs, NJ, USA) using a fiber optic coupler (Thorlabs, NJ, USA) to deliver a single-mode beam to the OR-PAM system. The beam from the fiber is collimated and focused by a reflective collimator and an objective lens (RC08FC-P01 and AC127-050-A, Thorlabs Inc., NJ, USA). The beam is focused on the target by a water-immersible micro-electro-mechanical system (MEMS) scanning module (OptichoM-MS, Opticho, Republic of Korea), which includes an opto-ultrasound beam combiner and a 1-axis MEMS scanning mirror (Fig. 1b). The scanning mirror operates from 30 Hz to 80 Hz. The PA waves generated on the target are returned to the beam combiner and acquired by a 50-MHz US transducer (V214-BC-RM, Olympus NDT, MA, USA) attached on the beam combiner. The acquired signals are amplified by a low-noise, high-gain amplifier (PE15A1013, Pasternack Enterprises, CA, USA) with a 50 dB gain. The amplified signals are digitized and transferred to a personal computer *via* a high-speed digitizer (ATS9350, Alazar Technologies, QC, Canada) with a 500 MS/s sampling rate. Additionally, two motorized linear stages (L-509, Physik Instrumente, Germany) are employed to expand the field of view (FOV). A multi-functional data acquisition (DAQ) board (PCIe-6321, National Instruments, TX, USA) used a fully synchronized timing sequence (Fig. 1c). The DAQ board generates a reference signal with the speed of the scanner. The trigger signals for the two lasers, digitizer, and scanner are initiated based on the timing of the positive edge of the reference signal. The 559 nm laser is started 4 μs late to prevent the PA signals from the two lasers from overlapping. The triggers of the lasers and digitizer for data acquisition are enabled only when the scanner scans unidirectionally. The data acquisition and storage were implemented and executed in LabVIEW (National Instruments, TX, USA), and post processing and data analysis were carried out in MATLAB (MathWorks, MA, USA).

The lateral and axial resolutions of the OR-PAM system are 5 and 30 μm, respectively [36]. This lateral resolution is sufficient to image single blood vessels in cuticles and fingers without blurring. The axial size of the blood vessels may appear slightly thicker due to the relatively poor axial resolution. The PA B-mode imaging speed of 50 Hz is sufficient catch the heart rate, the dominant frequency of the circulatory system. The step sizes along fast and slow scanning axes were 5 μm and 10 μm. The pulse repetition frequency was 40 kHz and 10 kHz for single- and multi-wavelength imaging, respectively. The signal-to-ratio (SNR), the maximum PA value of the blood vessels over the standard deviation of the background noise, was calculated to be 28 dB in human *in vivo* experiments described later.

### 2.2 *In vivo* PA imaging of human fingers with brachial cuffs

For human experiments, all experimental procedures were approved by the Institutional Review Board (IRB) of Pohang University of Science and Technology. We recruited a healthy volunteer as the subject of the *in vivo* experiments. Before the experiments, we completely explained the experimental procedure and received his informed consent. The operator and subject wore laser safety glasses and flame-retardant clothes for protection from the laser beam. For the experiments that required brachial pressure, the subject was first fitted with brachial cuff (REF CUF-F-A, A&D Company, Japan). Then, the subject’s right hand was placed on a customized finger holder fixed on a motorized vertical transition stage (8MVT188-20, Standa, Lithuania). After the little finger was covered with ultrasound gel, the vertical stage was moved to contact the finger against the thin membrane under the water tank. The water tank was then filled with enough water to submerge the MEMS scanning module. After the above preparation was completed, the subject took comfortable position, then the PA imaging experiments were performed. If brachial pressure was required, air was injected into the cuff using a digital blood pressure monitor (UA-651BLE, A&D Company, Japan). A pressure of over 150 mmHg was applied to ensure arterial occlusion. The measured laser pulse energies for the 532 and 559 nm beams were 530 nJ and 630 nJ, respectively, which correspond to 14.2 mJ/cm^2^ and 16.6 mJ/cm^2^, respectively, on the skin. Because the pulse widths of the beams are 10 ns and 18 ns, the conversion efficiencies for the beams from the pulse energy to the PA signal are different. To get the similar PA signal level from the two beams, we fixed the optical power of the 532-nm beam and adjusted the optical power of 559-nm beam by rotating the continuously variable ND filter wheel, while monitoring the PA signals on the blood vessels. The resulting optical exposures were below the maximum permissible exposure (MPE) level for skin, 20 mJ/cm^2^ set by the American National Standards Institute (ANSI) Z136.1 standard. We did not observe any laser-induced burns on the skin during or after the experiments.

## 3 Results and discussion

### 3.1 Photoacoustic monitoring of arterial pulsation

**Fig. 2:**
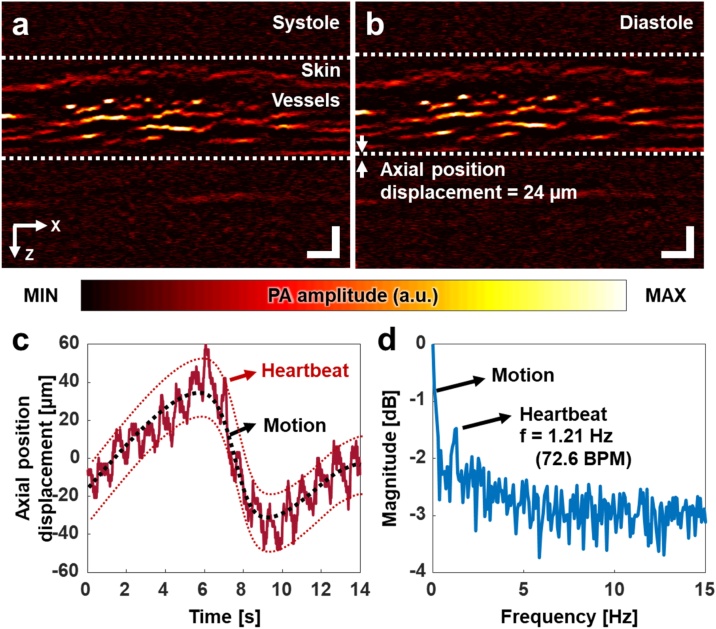
Photoacoustic monitoring of arterial pulsation based on the axial position displacement of the blood vessels in a finger. Photoacoustic B-mode of the finger during systole (a) and diastole (b). (c) Axial position displacement of the blood vessels over time. (d) Frequency response of the axial position displacement. The dominant frequency, 1.21 Hz, represents a pulsation at 72.6 beats per minute. All scale bars are 200 μm.

We continuously imaged a cross-sectional plane of the subject’s finger, at 50 Hz. Fig. 2a and b show PA B-mode images of the blood vessels during subsequent systole and diastole conditions, respectively. Supplementary Video S1 shows real-time PA monitoring of the systole and diastole dynamics. The vascular movement was quantified with following steps: (1) a region of interest (ROI) that includes the single blood vessel was selected in the first image. (2) A pixel that has the highest photoacoustic signal in the ROI was found. (3) The above steps were repeated on the consecutive images. (4) The pixels’ axial positions were accumulated. The axial position displacement over time shows heartbeat and motion (Fig. 2c). The blood vessels quickly oscillate with approximately 24 μm (red dotted lines in Fig. 2c) and slowly moves (black dotted line) by the heartbeat and motion, respectively. This movement by the heartbeat could be observed, because our PAM system has about 3-um axial step size from 500 MS/s sampling frequency, assuming that the speed of sound in water is 1500 m/s. The heartbeat exhibits a periodic movement with a frequency of 1.21 Hz (Fig. 2d), equivalent to 72.6 pulses per minute, and the motion has lower frequency components than those of the heartbeat. The periodic movement matches well with the subject’s heart rate as measured by a sensor on a smartphone (Galaxy Note9, Samsung Electronics, Republic of Korea). Thus, it can be concluded that the axial position displacement is considered as surrounding blood vessels' deformation caused by arterial pulsation located below the imaging area. Further, this consideration agrees well with the previously reported result using an ultrasound-array-based PAM and its validation using a commercial US machine [40].

### 3.2 Photoacoustic monitoring of blood perfusion after releasing a brachial cuff

**Fig. 3:**
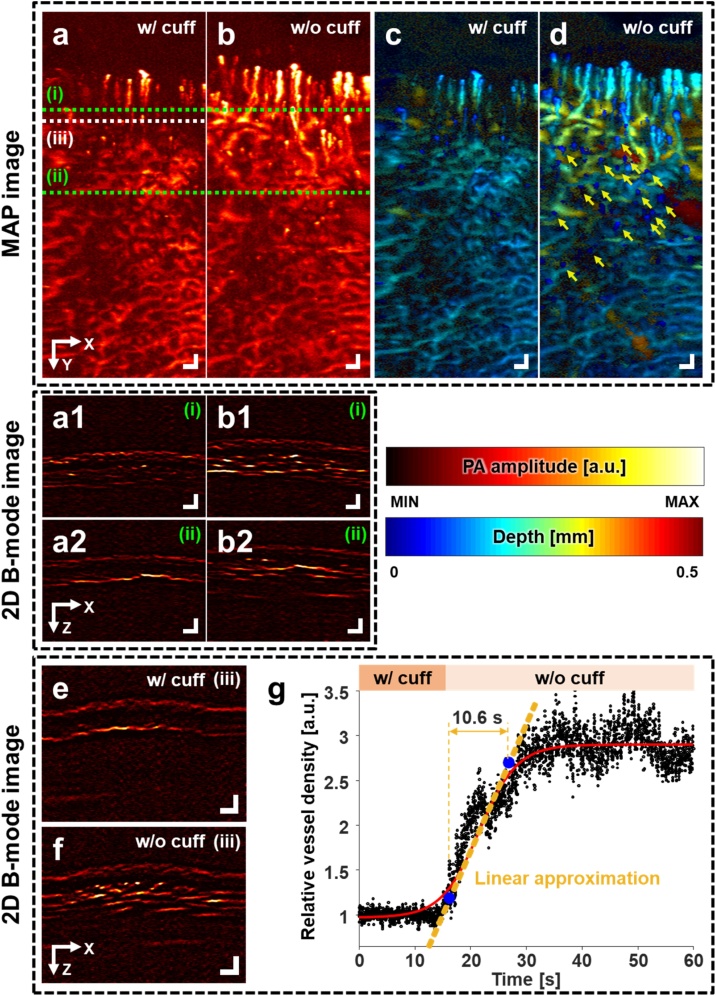
Real-time photoacoustic monitoring of blood perfusion in a finger during brachial cuffing and after release. PA MAP images (a) during and (b) after branchial cuffing. (a1, a2) PA B-mode images cut along lines (i) and (ii) in (a), respectively, and (b1, b2) PA B-mode images cut along lines (i) and (ii) in (b), respectively. (c, d) PA depth-encoded images of (a) and (b), respectively. Depth refers to the distance from the skin surface. (e, f) PA B-mode images with and without the cuff respectively, during continuous PA B-mode imaging near line (iii) in (a). (g) Relative change in vessel density over time. The cuff was released at 15 s. After the cuff is released, the vessel density increases for 10.6 s, and stays the same thereafter. All scale bars are 200 μm. PA, photoacoustic, and MAP, maximum amplitude projection.

To investigate dynamic changes, we continuously monitored the finger blood vessels and blood perfusion while releasing the brachial cuff. Fig. 3a and b are PA maximum amplitude projection (MAP) images of the finger during and after brachial cuffing, respectively. With the cuff, less blood vessels are observed than without the cuff. Fig. 3a1-2 and b1-2 are PA B-mode images cut along the green dashed lines (i) and (ii) in Fig. 3a, respectively. The difference in vascular density with and without cuff conditions is also confirmed in the cross-sectional B-mode images. Fig. 3c and d are PA depth-encoded images of Fig. 3a and b, respectively. After cuffing, petechiae, small areas of bleeding near the skin caused by the elevated blood pressure, are seen in Fig. 3d (yellow arrows). Representative PA B-mode images of the finger (along the white dashed line (iii) in Fig. 3a) with and without the cuff are shown in Fig. 3e and f, respectively. The change in vessel density is obvious when comparing these two B-mode images.

Additionally, we quantified the change in vessel density in Fig. 3g and Supplementary Video S2. Initially, the PA signals from the skin surface were removed using the skin profile estimator in 3D Photoacoustic Visualization Studio (3D PHOVIS) [41]. Then, after thresholding the PA B-mode images using a value four times higher than the noise level to clearly separate the PA signals from the blood vessels, we counted the number of filtered pixels. Finally, the relative vessel density was calculated by normalizing the number of the blood vessels without the cuff to that with the cuff (Fig. 3g). The blood vessels are about 2.8 times denser without the cuff than with the cuff. Further, we fitted the relative vessel density to the sigmoidal curve using a Boltzmann fitting, and the curve was linearly approximated at the center of the curve. The blood perfusion time is 10.6 s from the cuff release to recovery, measured by calculating the time difference between the 10 % and 90 % points of the linearized slope. These results are consistent with previously reported results on humans with arterial occlusion. This rapid and exaggerated reperfusion is reactive hyperemia that significantly reduces microvascular resistance and quickly supplies blood to oxygen-starved tissues in response to temporary ischemia [42]. The amount of reperfusion is proportional to the microvascular ability to dilate blood vessels, and reactive hyperemia predicts cardiovascular events because it evaluates impaired vasodilatory function [43].

A previously reported study on human palms [44] compared relative changes in the averaged value of PA signals on the capillary bed. This study could not investigate single blood vessels because the experimental instrument had low spatial resolutions, 70-μm lateral and 54-μm axial resolutions. Further, the low temporal resolution provided by the motor-driven B-mode imaging speed of about 1 Hz made it difficult to provide an accurate perfusion time. Compared to the previous study, our PAM's improvement on spatial and temporal resolutions enables to visualize single capillaries and observe vascular density in real time. We believe that we can estimate the blood perfusion time more accurately than in previous studies, thanks to our system's high resolution and high-speed imaging capability. A previous study [45] using the OCTA technique in the human nailfold with direct pressure, not a brachial cuff, observed a reduced signal from capillary regions and the occluded blood vessels when pressure was applied, and confirmed reperfusion after pressure was released. These OCTA results agree well with those of our PA imaging. However, while the OCTA study applied pressure directly to the skin area within the imaging FOV, we used brachial cuffing to block arterial and venous flows to prevent other tissues from being deformed. Other studies [46,47] have used using clinical PA/US systems to measure changes in PA signals from muscle and arteries/veins in the human forearm during the occlusion. Clinical systems can image deep tissues and vessels by sacrificing spatial resolution. Although these studies targeted larger blood vessels than we did, their results are consistent with ours: PA signals decreased during occlusion. In the future, the combination of the PAM and clinical PA/US systems, with their different imaging resolutions and depths [48], may allow us to better understand blood perfusion from microvessels to macrovessels.

Additionally, the high-resolution and high-speed PAM system could be a new tool for counting petechiae, such as those shown in Fig. 3d, during the tourniquet test for capillary fragility [49]. According to World Health Organization (WHO), the tourniquet test is considered positive for capillary fragility when 20 or more petechiae per 2.5 cm^2^ (1 inch square) can be observed by the naked eye [50]. In our case, over 20 petechiae per 2 mm^2^ can be seen, because the high-resolution volumetric PAM could detect small petechiae, invisible to the naked eye, on and under the skin. Compared to conventional visual examination, high-resolution and high-speed capabilities enable the PAM to detect up to 10-um petechiae in seconds, both on the skin and under the skin. We found that the petechiae on and under the skin can be photoacoustically detected, which suggests that the high-resolution and high-speed PAM system could be a new quantification tool to count the number of the petechiae during the tourniquet test.

### 3.3 Change in oxygen saturation during arterial occlusion

**Fig. 4:**
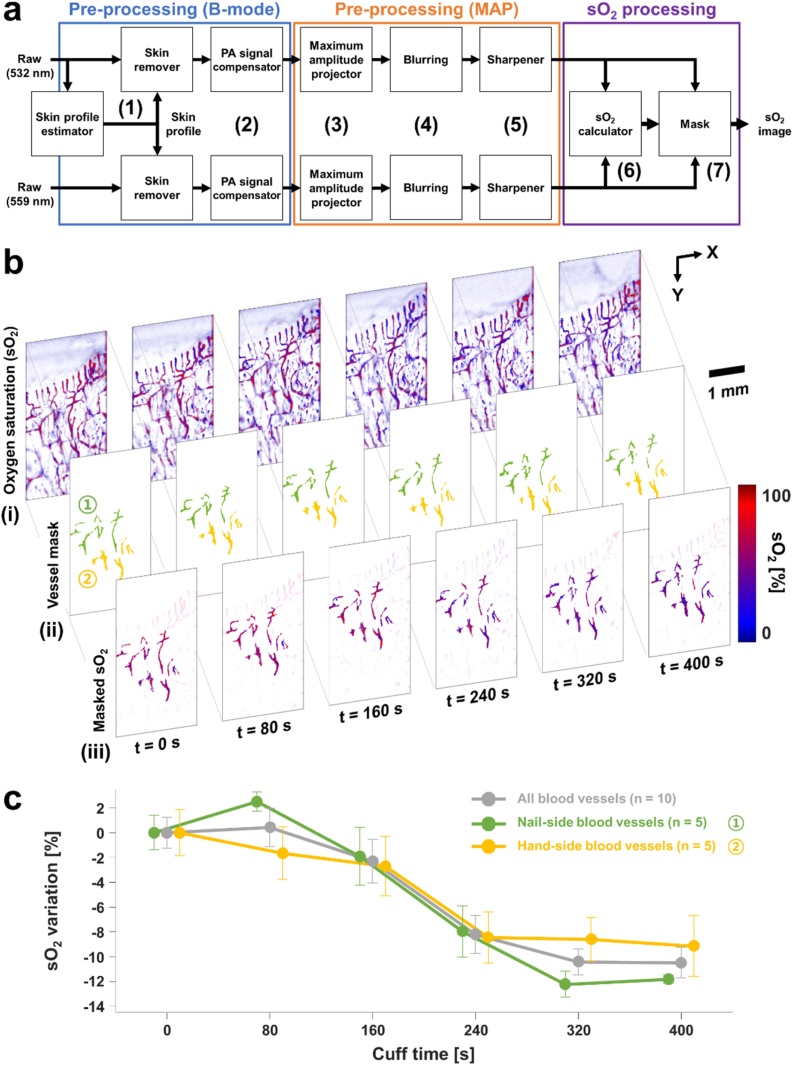
Change in oxygen saturation (sO_2_) in a finger due to arterial occlusion. (a) sO_2_ processing procedure. (b) Consecutive (i) sO_2_, (ii) vessel mask, and (iii) masked sO_2_ images during 400 s with a brachial cuff. The blue and red vessel masks indicate nail- and hand-side blood vessels, respectively. (c) Change in sO_2_ values of the nail- and hand-side blood vessels over time. The error bar represents the standard error of the averaged sO_2_ of the blood vessels in the vessel masks.

To investigate the functional imaging capability of our system, we monitored the sO_2_ change in finger vessels during arterial occlusion. Six consecutive sets of volumetric PA images were acquired, over 400 s, at 532 and 559 nm. The three steps of the sO_2_ image and signal processing procedures are shown schematically in Fig. 4(a). In the first step, the PA B-mode images were pre-processed as follows: (1) The skin layer on the raw PA data was removed using the skin profile estimator in 3D PHOVIS. (2) The PA signal levels from focused and unfocused regions were equalized, using the background PA signals to compensate for optical and acoustic attenuation [51]. (3) The maximum values at each position were projected to get PA MAP images. In the second step, the PA MAP images were pre-processed as follows: (4) The PA MAP images were blurred to minimize differences in the PA signals due to laser fluctuation and MEMS scanning. (5) To resharpen the blood vessels’ boundaries, a high-pass filter was applied to the blurred images. In the third and final step, we used spectral unmixing to obtain the PA sO_2_ image [52]. (6) The sO_2_ values were calculated pixel by pixel. (7) Using the mask created by thresholding the PA MAP images, the valid sO_2_ values were extracted and made into a PA sO_2_ image.

Fig. 4b(i) shows consecutive PA sO_2_ images during arterial occlusion. The overall color of the images gradually changes from red to blue, indicating decreasing sO_2_ values due to the curtailed oxygen supply. In the PA sO2 images, we selected big blood vessels with high SNR for reliable analysis. For blood vessels that are small or out of focus, the variability of their sO2 values was severe due to the low SNR, so these blood vessels were excluded from the analysis. To extract reliable sO_2_ values, we identified well-connected vessels using the connected-component labeling function in the Image Processing Toolkit in MATLAB, and then we manually picked out ten blood vessels [53]. The selected blood vessels were classified into two groups, nail-side and hand-side, and used as a mask to obtain vessel-masked PA sO_2_ images (Fig. 4b(ii) and (iii)). We calculated the mean and standard error of the averaged sO_2_ values of each blood vessel group. The sO_2_ values of the nail- and hand-side blood vessels decreased to 11.8 % and 9.2 % from the baseline, respectively, with the same slope (Fig. 4c). In this experiment, we quantitatively confirmed that the finger continued to consume oxygen during the arterial occlusion. The rate of sO_2_ decrease became smaller at approximately 5 min after the arterial occlusion. This saturation phenomenon was also observed in a previous study [44], which reported the relative PA signal ratio during arterial occlusion using wavelengths of 561 and 570 nm, on which deoxy- and oxy-hemoglobin have higher absorption coefficient than the other wavelength, respectively. The amount of the sO_2_ variation is similar to the sO_2_ difference measured in animals during the physiological states of normoxia and hypoxia [54].

A previously reported study used near-infrared spectroscopy (NIRS) imaging [55] to measure sO_2_ changes in human hands. The regional sO_2_ change in tissues during arterial occlusion showed a similar trend to that of the blood vessels in our study. However, because NIR light scatters in the tissue volume as it travels from source to detector [56], NIRS imaging cannot separate the effects of different tissues and visualize blood vessels, so it simply provides an average sO_2_ value for a specific area. Despite its relatively poor resolution, NIRS imaging is relatively unaffected by the subject’s motion, which enables long monitoring, including before, during, and after an occlusion. In our study, on the other hand, we could continuously monitor single blood vessels and their sO_2_ only during the occlusion. Due to unintended motion at the moment of occlusion or release, it was difficult to maintain the same finger position and to continue monitoring the same blood vessels before, during, and after the occlusion. To minimize the effect of inevitable motions, a finger-mounted structure such as a fingertip pulse oximeter could be an alternative. By capitalizing on the different principles and strengths of PA and NIRS, dual mode imaging techniques can provide sO_2_ information from capillaries and local tissues, advancing our understanding of the microcirculation.

## 4 Discussion and conclusion

Using high-resolution, high-speed, and spectroscopic PAM, we obtained quantitative physiological information about blood vessels in human fingers *in vivo*. We also extracted the heart rate from the axial position displacement of the blood vessels. Currently, photoplethysmography (PPG) is frequently used to obtain the heart rate [57], directly measuring variations in blood volume caused by arterial pulsation [58]. Pursuing a different approach, the PA imaging in our study indirectly extracted the axial position displacement induced by arterial pulsation. Because arterial volume variations and the accompanying displacement of the surrounding blood vessels are interrelated, analyzing these physical changes requires simultaneous PA imaging and PPG sensing. In addition, the amount of the axial position displacement, such as the 24 μm shown in Fig. 2, may be related to the amplitude of the PPG signals and/or blood pressure.

During the arterial occlusion, the perfusion time and the sO_2_ variation were also investigated. These results for the perfusion time and sO_2_ variation are consistent with those in previous non-imaging-based studies. In addition, previous clinical NIRS studies also showed similar results for perfusion time and sO_2_ variation in healthy volunteers, although not providing imaging results [59,60]. From a wider perspective, by using multiple wavelengths, the PPG technique can also estimate the sO_2_ in arteries [61]. PA imaging and PPG sensing provide the non-arterial and arterial sO_2_, respectively, so knowing the sO_2_ in different types of blood vessels at a time would be useful in analyzing oxygen transfer in the blood vessels. Furthermore, the combination of the above-mentioned imaging and sensing techniques with PA, PPG and NIRS could help understand the blood circulation on both macroscopic and microscopic levels. In addition, the simultaneous imaging with multiple sources implemented in this paper allows multi-resolution imaging (*e.g.*, optical- and acoustic-resolution PA imaging) and/or multi-modal imaging (*e.g.*, US/PA imaging), which can visualize deeper blood vessels or surrounding anatomical structures [62,63]. The PAM could be a vascular visualization tool for peripheral vascular disease because it provides vascular and functional imaging. In addition to the applicability of the PAM itself, it can be easily combined with optical and ultrasonic technologies. The PAM study using a transparent ultrasonic transducer [64] is accelerating the combination of these technologies, which would help to diagnose PAD by providing multiple clinical information.

In conclusion, we visualized and measured multiparametric information about human finger microvessel networks *in vivo,* using high-resolution, high-speed, spectroscopic PAM. The high-resolution and spectroscopic capabilities enabled visualizing single blood vessels and measuring the sO2 in the blood vessels. With the cost-effective configuration of the single US transducer and MEMS scanner, the high-speed imaging capability of the PAM system made it possible to measure the clinical indicators, the heart rate, perfusion time, and sO_2_ variation. These results show that vascular dynamics and some clinical indicators can be measured and evaluated *via* photoacoustic monitoring. Nevertheless, several improvements are easily envisioned. Either finger-mounted or handheld devices, or perhaps auxiliary tools would allow conducting experiments in a comfortable position that minimizes inevitable motions [[65], [66], [67]]. The heartrate and vascular density change were quantified on 2D images due to slow 3D imaging speed. However, these in-plane quantification may be inaccurate because it is affected by out-of-plane motions. For more reliable results, it is necessary to develop better laser sources with faster repetition rates, less fluctuation, and more wavelengths. Motion correction can be applied to mitigate the inevitable motions [68,69], and vascular enhancement can be adopted to achieve high-quality blood vessel images [70,71]. From the improvement, if real-time 3D imaging is available, the heartbeat and vascular density could be more accurate. Further investigations of the pressure, position, and application time of the brachial cuff for occlusion are needed to obtain clinically meaningful results. With such technical improvements, it is essential to conduct comparative clinical studies on the proposed vascular functions at the distal ends between different groups. (*e.g.*, young *vs*. elderly or patients *vs*. controls). In the future, the high-resolution, high-speed, spectroscopic PAM could provide more reliable physiological results and become a fully realized tool for screening, diagnosing, and predicting the progress of vascular conditions.

## Declaration of Competing Interest

Chulhong Kim and Jin Young Kim have financial interests in OPTICHO, which, however, did not support this research.
